# Multifunctionalized biocatalytic P22 nanoreactor for combinatory treatment of ER+ breast cancer

**DOI:** 10.1186/s12951-018-0345-2

**Published:** 2018-02-20

**Authors:** Kanchan Chauhan, Juan M. Hernandez-Meza, Ana G. Rodríguez-Hernández, Karla Juarez-Moreno, Prakhar Sengar, Rafael Vazquez-Duhalt

**Affiliations:** Department of Bionanotechnology, Centro de Nanociencias y Nanotecnología, Universidad Nacional Autónoma de México, Km. 107 carretera Tijuana-Ensenada, 22860 Ensenada, Baja California Mexico

**Keywords:** Cytochrome P450, Photodynamic therapy, Reactive oxygen species, Tamoxifen, Virus-like particle

## Abstract

**Background:**

Tamoxifen is the standard endocrine therapy for breast cancers, which require metabolic activation by cytochrome P450 enzymes (CYP). However, the lower and variable concentrations of CYP activity at the tumor remain major bottlenecks for the efficient treatment, causing severe side-effects. Combination nanotherapy has gained much recent attention for cancer treatment as it reduces the drug-associated toxicity without affecting the therapeutic response.

**Results:**

Here we show the modular design of P22 bacteriophage virus-like particles for nanoscale integration of virus-driven enzyme prodrug therapy and photodynamic therapy. These virus capsids carrying CYP activity at the core are decorated with photosensitizer and targeting moiety at the surface for effective combinatory treatment. The estradiol-functionalized nanoparticles are recognized and internalized into ER+ breast tumor cells increasing the intracellular CYP activity and showing the ability to produce reactive oxygen species (ROS) upon UV_365 nm_ irradiation. The generated ROS in synergy with enzymatic activity drastically enhanced the tamoxifen sensitivity in vitro, strongly inhibiting tumor cells.

**Conclusions:**

This work clearly demonstrated that the targeted combinatory treatment using multifunctional biocatalytic P22 represents the effective nanotherapeutics for ER+ breast cancer.

**Electronic supplementary material:**

The online version of this article (10.1186/s12951-018-0345-2) contains supplementary material, which is available to authorized users.

## Background

Breast cancer is the most common malignancy and the primary cause of mortality among women worldwide. Approximately 80% breast cancer cases are estrogen receptor positive (ER+) and often respond to endocrine therapy using tamoxifen as an antiestrogen [1, 2]. Tamoxifen is a classic pro-drug that involves the metabolic activation by the catalytic action of a family of cytochrome P450 enzymes (CYP) to elicit pharmacological activity [3, 4]. However, CYP activity is greatly dependent on both genetic as well as environmental (drug-induced) factors that contribute to variable therapeutic response in interindividuals [2]. Moreover, breast tumors have lower CYP concentrations and thus, the effective drug dose is only a fraction of administered drug. Consequently, repetitive drug administration is required leading to severe side-effects associated with hepatic dysfunctions and diseases [5, 6]. There still exist the major obstacles including the ability of this cancer to adapt, evolve and become resistant to the treatment strategies. Nevertheless, with the recent advancements in the early detection of breast carcinoma, new modalities to treat this cancer at all the stages will be advantageous.

Cancers are heterogenic and complex diseases that involve multiple physiologies thus, multi-targeting using combination therapy is at the forefront of research against cancer that offers improved therapeutic response with reduced drug dose and resistance [7–12]. Nevertheless, the effective drug administration based on the dissimilar biodistribution, pharmacokinetics and toxicity profile is a complicated process, and could be well addressed via combination nanotherapy. Over the past two decades, a number of nanoparticles have emerged for a wide variety of nano-medicinal applications including the delivery of multiple drugs for combination therapy [13, 14]. Challenges remain in the toxicity, ability to overcome biological barriers and biodistribution of many of these nanoparticles, resulting from their low biocompatibility and small size. Unlike polymer-based, liposomal or metal-based nanoparticles, organized protein-based nanomaterials, such as virus-like particles (VLPs) have the potential to address these challenges by providing a biocompatible scaffold and enabling control over the shape and size of the structures. These robust VLPs are versatile biomacromolecular structures that are highly resilient against the biological insults and represent the effective nanocarriers for enzymes [15–17]. In our previous work, VLPs derived from bacteriophage P22 were developed as a nano-bioreactor encapsulating an enhanced peroxygenase, CYP_BM3_ from *Bacillus megaterium*, for virus-driven enzyme prodrug therapy (EPT) [18–20]. The 53 nm icosahedral P22 nanoreactor assembled 420 coat protein (CP) with 109 copies of the CYP_BM3_-scaffold fusion protein [19]. The biocatalytic nanoparticles were functionalized to be recognized and internalized into tumor cells increasing the CYP activity for maximum pro-drug transformation and reducing the drug doses [20].

The VLP’s are also proven as optimal nanoplatforms for photodynamic therapy (PDT), a clinically approved non-invasive treatment modality [21–23]. PDT utilizes a photosensitizer (PS) moiety, which produces reactive oxygen species (ROS) when activated by the irradiation at its resonance wavelength causing lesion destruction. The potential of PDT combination with other conventional therapies has been recognized as a strategy to improve the therapeutic efficiency of treatments in modern oncology [24–26]. Because the PDT combined therapies are confined to the illuminated area, the potentiated toxicity is not systemic. This is especially important in elderly or debilitated patients who poorly tolerate intensive therapeutic programs. PDT can be safely combined with other antitumor treatments without the risk of inducing cross-resistance. Moreover, promising new approaches that include the tumor targeting by the use of nanoparticles has been recently reviewed [27, 28]

The combination of biocatalytic P22 with a photosensitizer via genetic and chemical engineering strategies will allow the combination of EPT and PDT at a common nanoplatform for combinatorial nanotherapy as well as theranostics. The selective targeting of such multimodal system will specifically hit the tumor tissue by multiple pathways at once and diminish the drug-associated side-effects leading to better quality of patient’s life. Estrogen receptor (ER) belongs to the hormone receptor family and mainly localized on the cell membrane and intracellularly [29, 30]. It possesses a very high affinity towards its cognate ligands (estrogens) and thus, estradiol based ligands have been widely used as selective targeting agents for breast cancer imaging and therapy [31, 32].

In the present work, ER+ targeted VLPs performing the combination of EPT and PDT for synergistic cytotoxicity on breast tumor cells is demonstrated. As far we know, this is the first time in which a multifunctional nanoparticle able to enzymatic pro-drug activation, to produce ROS, and to be specifically targeted to tumor cells have been designed and tested. The biocatalytic P22 (P22CYP) is multifunctionalized with a well-known PS, protoporphyrin IX (PpIX) and an estradiol based targeting ligand. The VLPs retained their physical attributes after chemical modification and enabled ligand-mediated tumor targeting, enhancing the intracellular CYP activity and PS payload. Most importantly, the synergy between the two modalities using multifunctionalized biocatalytic VLPs not only improved the tamoxifen sensitivity, but also significantly enhanced the PDT response in MCF-7 cells. These preliminary analyses provide the meaningful steps towards the translatable medical applications of VLP technology.

## Results

### Synthesis

**Fig. 1:**
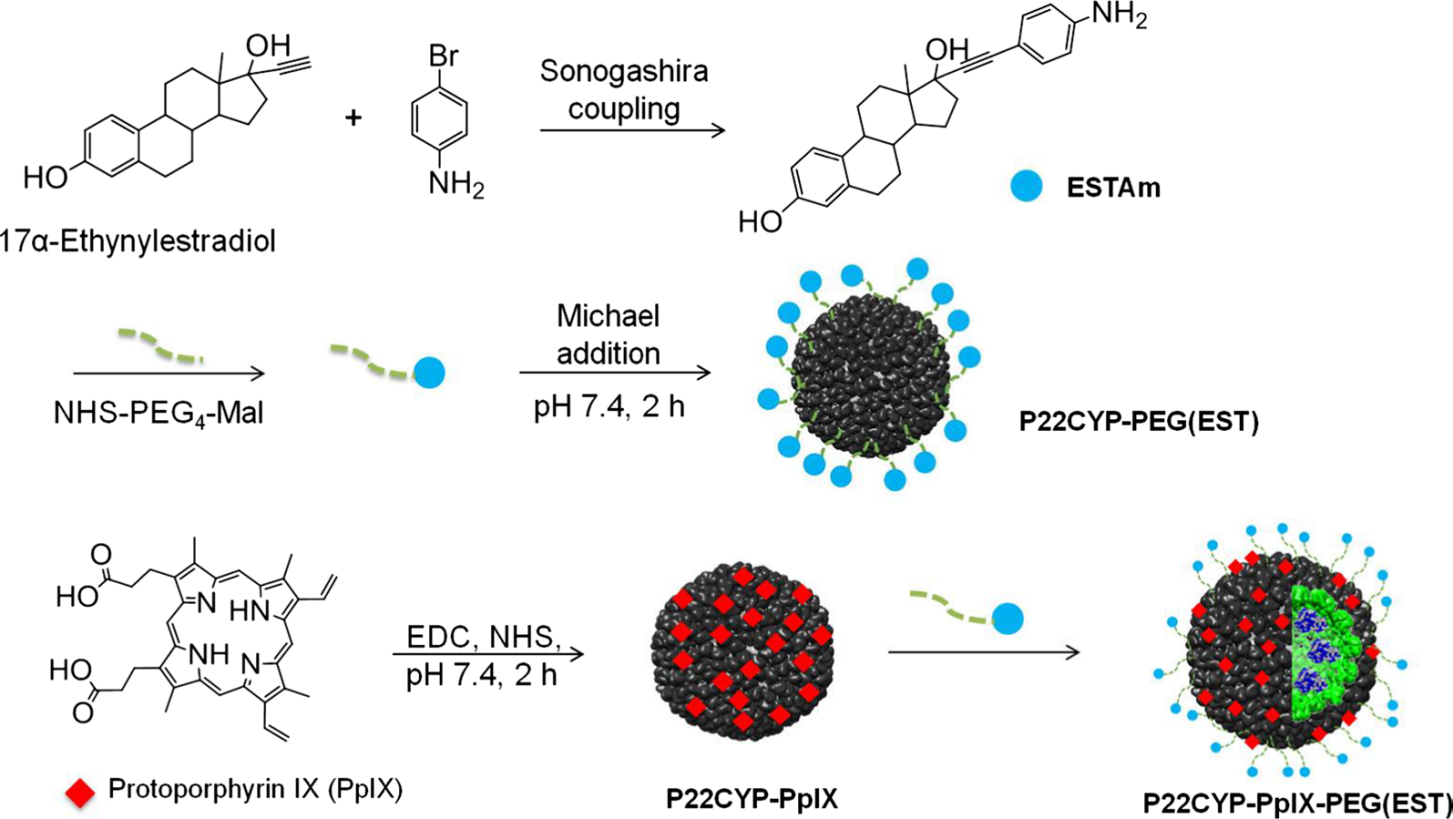
Schematic representation for synthesis of multi-functionalized P22CYP-PpIX-PEG(EST)

The design of the multifunctional VLP started with the self-assembly of P22CYP as described earlier [19, 20]. To endow P22CYP with PS, protoporphyrin (PpIX) is coupled to the surface exposed amine groups via carbodiimide chemistry giving P22CYP-PpIX (Fig. 1). Selective targeting to tumor cells was obtained by the functionalization of P22CYP with polyethylene glycol (PEG) containing an estradiol derivative, ESTAm, synthesized from the Sonogashira coupling of 17α-ethynylestradiol (EE2, an estrogen derivative) and 4-bromoaniline. Synthesis of ESTAm was confirmed by NMR, HRMS and FTIR analysis (Additional file 1: Figures S1–S3). A hetero-functional PEG with succinimidyl ester and the maleimide group (NHS-PEG_4_-Mal) at the distal ends was used. It enabled the selective conjugation of ESTAm via amide bonding giving PEG(EST), while maleimide site was open for further conjugation with nanoparticles. Finally, the Michael addition of PEG(EST) with the surface exposed amine groups of P22CYP and P22CYP-PpIX at pH 7.4 afforded targeted particles, P22CYP-PEG(EST) and P22CYP-PpIX-PEG(EST) respectively (Fig. 1 and “Methods”).

### Characterization

**Fig. 2:**
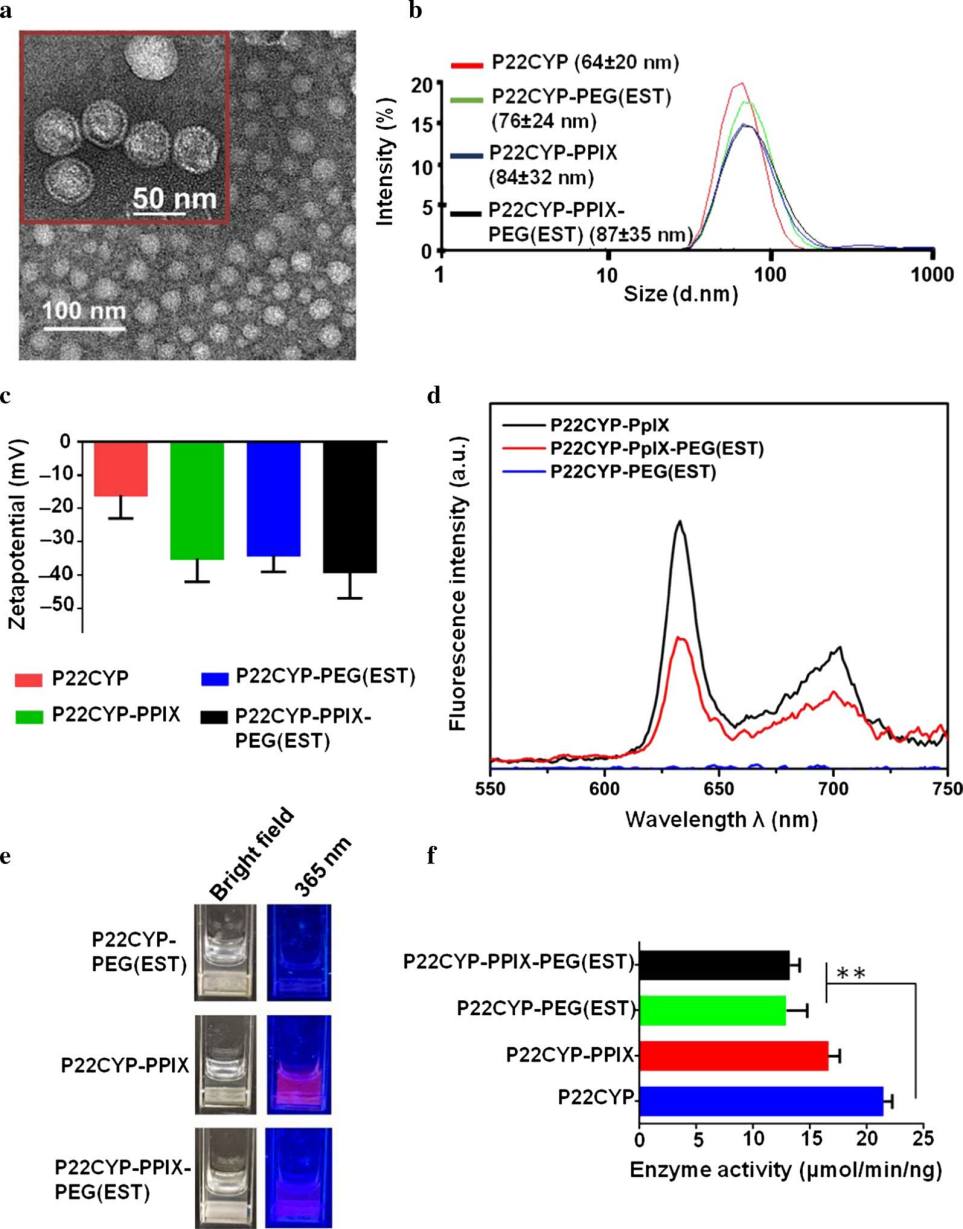
Characterization of P22CYP and functionalized P22CYP biocatalytic nanoparticles. **a** TEM micrographs of P22CYP-PpIX-PEG(EST) at different magnification. **b** Size distribution of P22CYP and functionalized P22CYP by DLS analysis. **c** Variation of surface charge of P22CYP and functionalized P22CYP by zeta potential analysis. **d** Photoluminescence analysis of functionalized nanoparticles at λ_ex_ = 405 nm. **e** Visualization of designed nanoparticles under bright light and UV_365 nm_. **f** Variation in enzyme catalytic activity after P22CYP functionalization. Results are expressed as the mean ± SEM (n = 3). **p < 0.01 using a one-way ANOVA with Tukey posttest

The TEM micrographs and dynamic light scattering (DLS) displayed the intact monodisperse particles after complete modification (Fig. 2a, b). The DLS analysis in de-ionized water revealed the hydrodynamic diameter (Dz) of P22CYP as ~ 64 nm, which increased to ~ 87 nm in P22CYP-PpIX-PEG(EST) (Fig. 2b). The zeta potential analysis displayed a significant increase in the surface negative charge from P22CYP (− 16 ± 6.7 mV) to P22CYP-PpIX-PEG(EST) (− 39 ± 7.7 mV) probably due to the reflecting interactions of the attached groups with aqueous medium (Fig. 2c). Further, the fluorescence analysis of functionalized particles was performed at room temperature under the PpIX excitation, λ = 405 nm. The designed nanoparticles showed the characteristic emission peaks of PpIX at 635 and 725 nm in the samples containing PpIX, and it was absent in P22CYP-PEG(EST) (Fig. 2d). The functionalization of P22CYP-PpIX with PEG(EST) led to the quenching of emission intensity, possibly due to the PEGylation of the particle surface. Consistently, the observation of P22CYP-PpIX under UV_365 nm_ (one of the absorption wavelength of PpIX) displayed the distinct red fluorescence of PpIX, which decreased in P22CYP-PpIX-PEG(EST) due to pegylation and was found absent in P22CYP-PEG(EST) (Fig. 2e). The ROS generation capability of conjugated PpIX was confirmed when P22CYP-PpIX-PEG(EST) was mixed with the ROS sensitive dye, 1,3-diphenyl isobenzofuran (DPBF) and irradiating with UV_365 nm_ (Additional file 1: Figure S4). Finally, the catalytic activity of VLP’s after the chemical modification was analyzed with 2,6-dimethoxyphenol. The CYP catalytic activity of P22CYP-PpIX was ~ 80% of the P22CYP nanoparticles, while the PEGylated particles, P22CYP-PpIX-PEG(EST) and P22CYP-PEG(EST), showed ~ 62% (Fig. 2f).

### Ligand-mediated intracellular localization

**Fig. 3:**
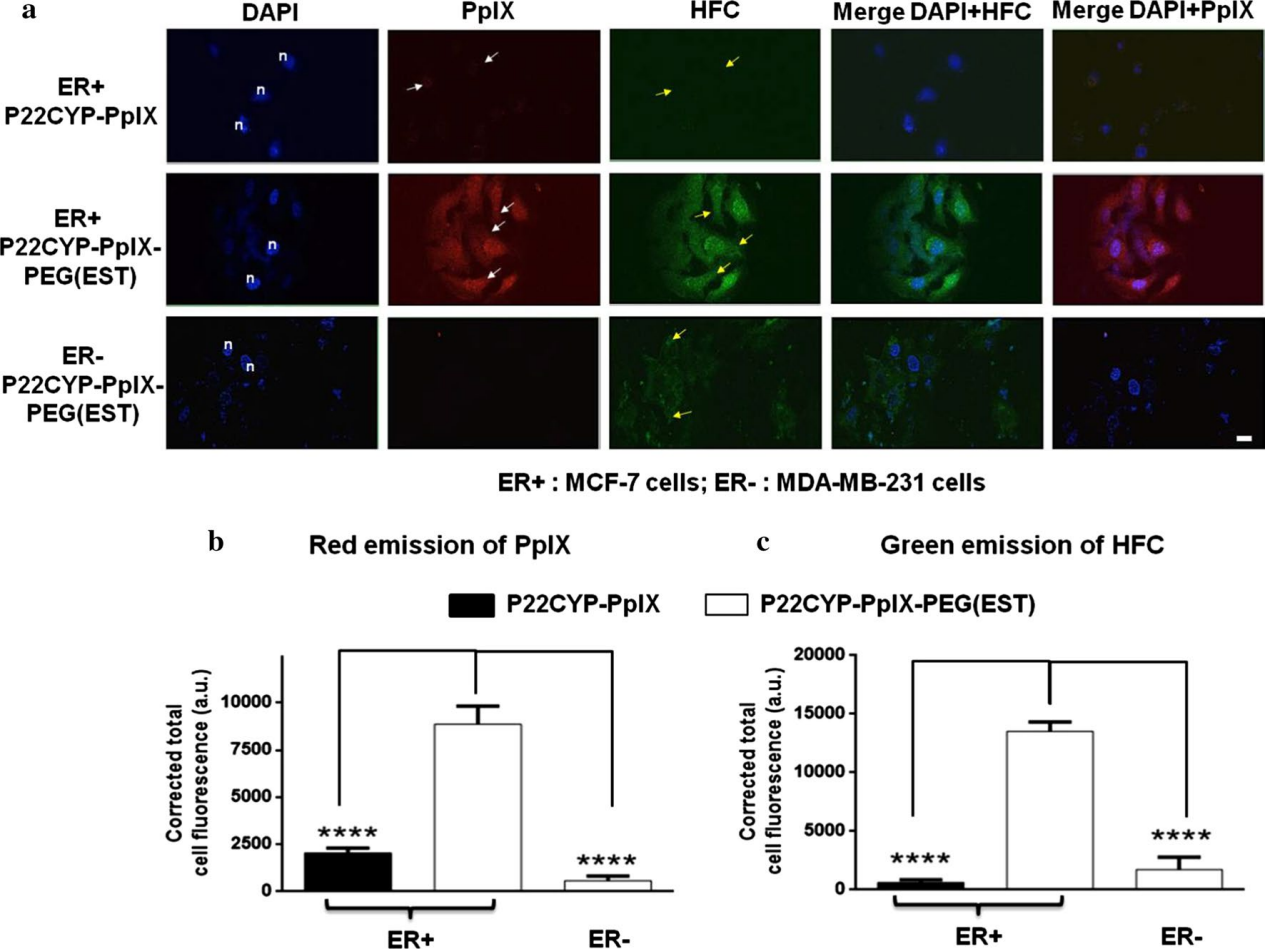
Preferential uptake of P22CYP-PpIX-PEG(EST) in MCF-7 cells. **a** Confocal fluorescence images of MCF-7 and MDA-MB-231 cells treated with P22CYP-PpIX (control) and P22CYP-PpIX-PEG(EST) and counterstained with DAPI where ‘n’ shows the stained nuclei of cells. The delivered CYP activity was measured by the transformation of BFC substrate into the green fluorescent HFC. The localization of PpIX and HFC to the cytoplasm of cells is shown with the white and yellow arrows respectively. The mean normalized fluorescence intensity of **b** PpIX red emission, and **c** HFC green emission per cell was quantified by ImageJ software. Results are expressed as the mean ± SEM (n = 3). ****p < 0.0001 using a one-way ANOVA with Dunnett’s posttest

The intracellular delivery of functionalized particles P22CYP-PpIX and P22CYP-PpIX-PEG(EST) was evaluated in MCF-7 (ER+) and MDA-MB-231 (ER−) human breast tumor cells by using confocal microscopy and monitored at the red fluorescence of PpIX. These cell lines were chosen due to the significant difference in the estrogen receptor (ER) expression [33]. The VLP internalization was also corroborated by the intracellular CYP delivery and analyzed by measuring the CYP activity via enzymatic transformation of the specific substrate, benzyloxy-4-trifluoromethylcoumarin (BFC), to the green fluorescent product, 7-hydroxy-4-trifluoromethyl-coumarin (HFC). After 12 h culture with nanoparticles, the ER+ cells showed the preferential intracellular and endosome localization of P22CYP-PpIX-PEG(EST) particles (Fig. 3a) as evident by the significantly higher intensity of PpIX red emission (Fig. 3b) and HFC green emission (Fig. 3c) when compared to P22CYP-PpIX. These findings reveal the ability of estradiol derivative as a ligand to greatly facilitate the cellular internalization. As expected, the P22CYP-PpIX-PEG(EST) did not show apparent cellular internalization in ER− cell lines, demonstrating the high specificity of ligand-functionalized particles.

### Intracellular ROS measurement after UV_365 nm_ irradiation

**Fig. 4:**
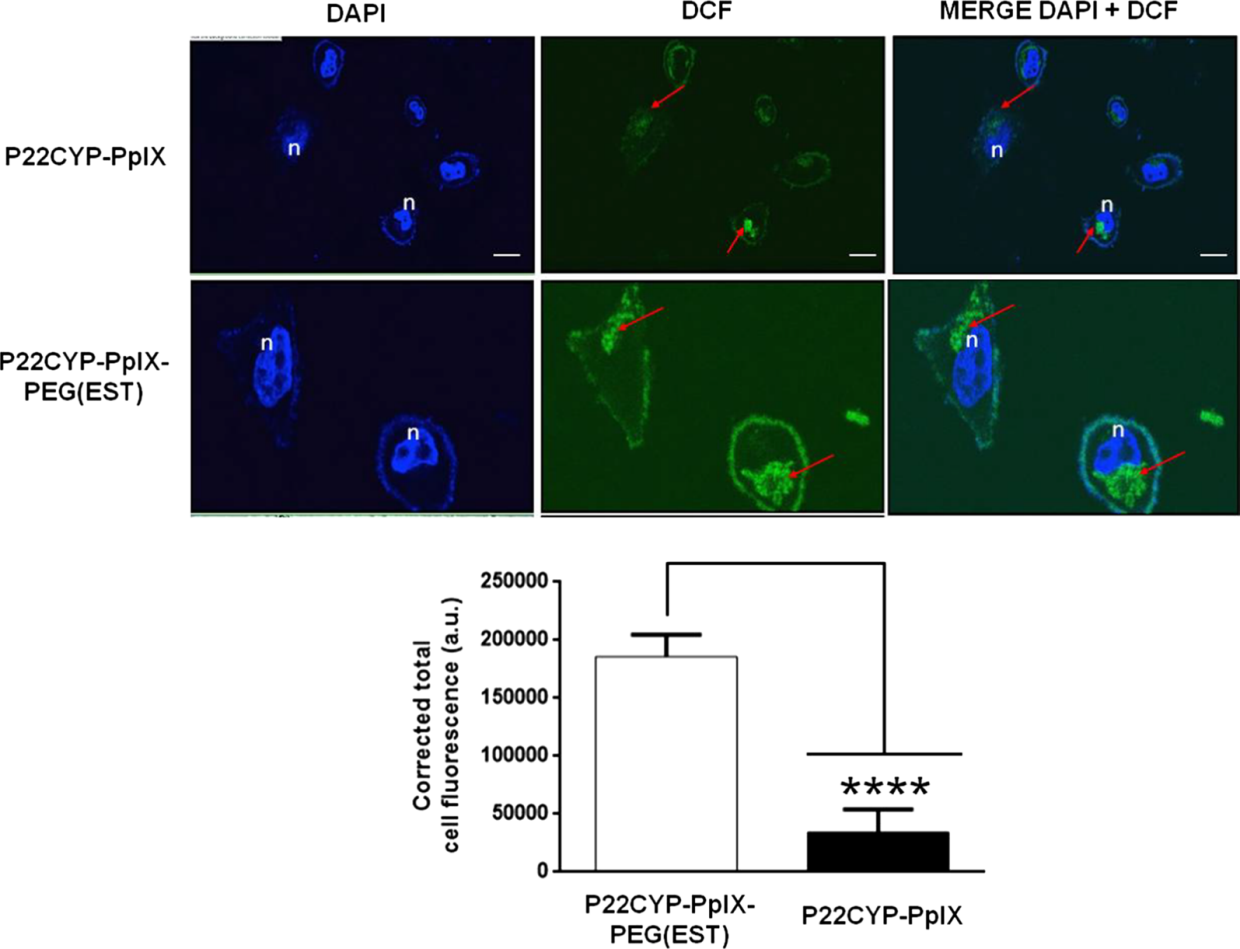
Activation of functionalized PpIX in P22CYP-PpIX-PEG(EST) by UV_365 nm_ (3 J/cm^2^) produced effective ROS. Cells were grown and incubated with the particles for 12 h, washed with PBS, treated with DCFDA for 30 min and then photo-irradiated. Green emission of DCF was analyzed by confocal microscopy and quantified by ImageJ software. Results are expressed as the mean ± SEM (n = 3). ****p < 0.0001 using Sudent’s *t* test

The efficiency of PDT is largely dependent on the ROS production after activation of photosensitizer by light. Thus, the intracellular ROS generation efficiency of P22CYP-PpIX and P22CYP-PpIX-PEG(EST) was analyzed in MCF-7 cells on the basis of green fluorescence of 2′,7′-dichloro-fluorescein (DCF) by confocal microscopy. Besides the quenching of PpIX emission in P22CYP-PpIX-PEG(EST) due to PEGylation, the intracellular ROS content in cells treated with these nanoparticles showed significantly higher green fluorescence of DCF than P22CYP-PpIX, and they seem to be located in endosomes (Fig. 4).

### Selective estrogen cell targeting

**Fig. 5:**
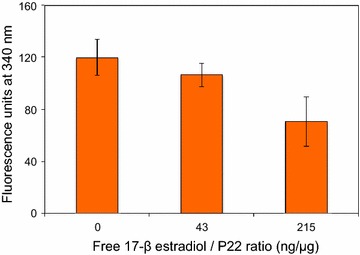
Ligand-receptor competition assay. The specificity of P22CYP-PpIX-PEG(EST) to be internalized in MCF-7 cells was evaluated by a competition assay using different ratios of VLP and free 17β-estradiol. MCF-7 cells (10,000 cells/well) were incubated with and without 17β-estradiol (0, 43 and 215 ng/μg of VLP protein) and the cell internalization was estimated by the transform BFC into HFC. The fluorescence intensity was measured with an excitation at 280 nm and the maximal emission was measured at 340 nm. The endogenous CYP activity was determined in tumor cell cultures without the addition of nanoparticles and subtracted from the treatments

In order to prove that the estradiol moiety of functionalized nanoparticles acts as ligand for specific receptors, a competition experiment was carried out. The specificity of P22CYP-PpIX-PEG(EST) to bind to MCF-7 cells was evaluated by a competition assay using two different ratios of VLP and free 17β-estradiol (Fig. 5) and by measuring their cell internalization by capability to transform BFC into HFC. The presence of increasing concentration of free 17β estradiol significantly reduces the nanoparticle cell internalization, demonstrating a competition for the estradiol receptors in the tumor cell surface. The presence of 0.215 μg of free estradiol per μg of protein of P22CYP-PpIX-PEG(EST) in the cell culture reduced to 58% the fluorescence originated by the nanoparticle CYP activity. Higher estradiol concentrations induced a detachment of the cells.

### Cytotoxicity assay

**Fig. 6:**
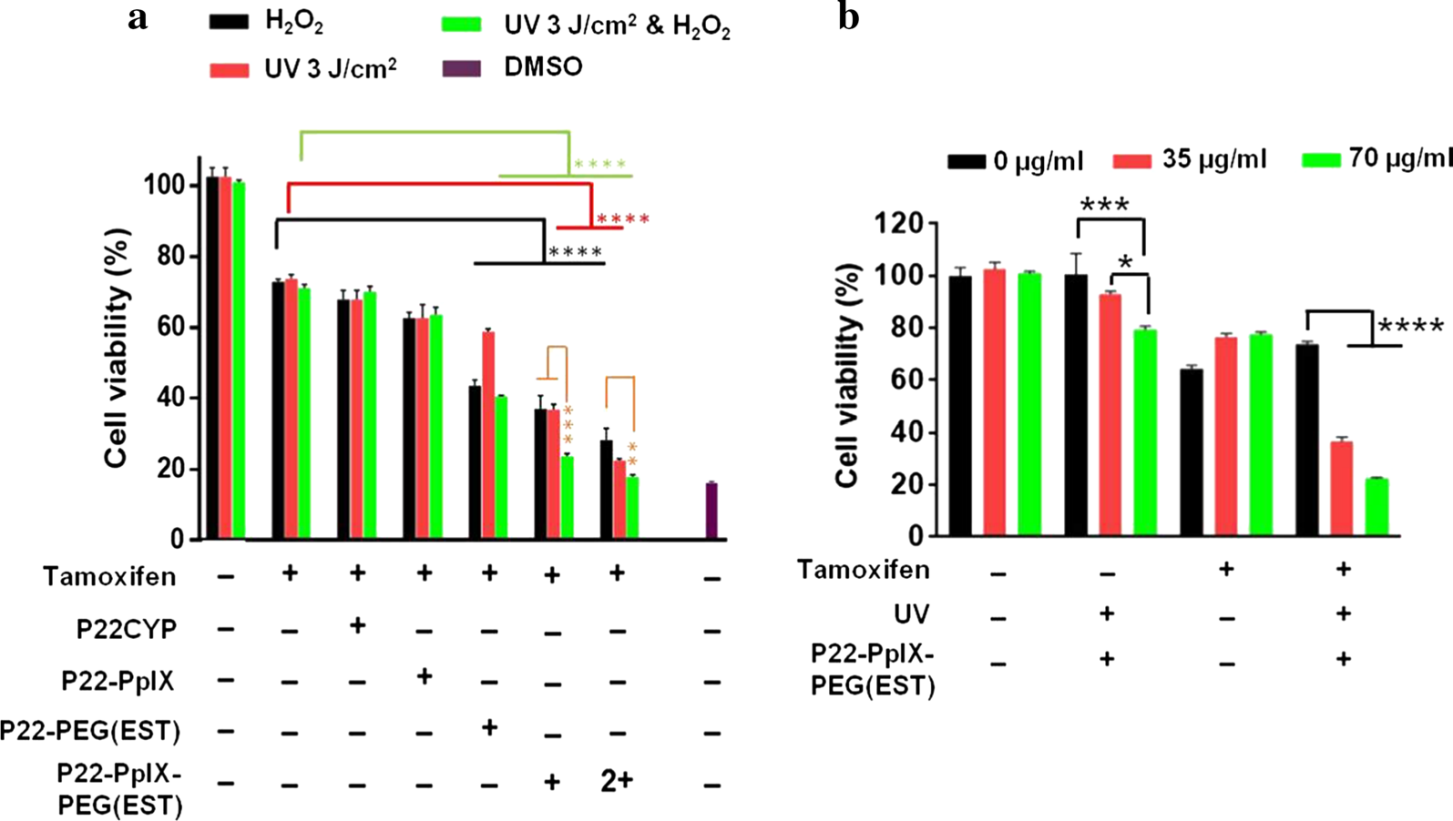
Effect of treatment with P22CYP and functionalized VLPs on MCF-7 cells sensitivity to tamoxifen by MTT assay. **a** Cell viability after an individual (EPT and PDT) and combinatory therapy where functionalized PpIX was activated by the irradiation of UV_365 nm_ (3 J/cm^2^) and encapsulated CYP was activated by H_2_O_2_ (3 mM) treatment. **b** Effect of different concentrations of P22CYP-PpIX-PEG(EST) on cell viability in the absence and presence of UV irradiation and/or tamoxifen. Results are expressed as the mean ± SEM (n = 3). *p < 0.05, **p < 0.01, ***p < 0.001, ****p < 0.0001 using a two way ANOVA with Tukey post test

The cell viability of MCF-7 cells treated with tamoxifen in the presence and the absence of biocatalytic VLPs was determined. The experiment was designed to discriminate the effect of EPT and PDT separately, and the combination of both. Preliminarily, the dose dependent toxicity of tamoxifen on MCF-7 cells was assayed (Additional file 1: Figure S5). A cell viability of > 70% was obtained in the presence of 20 μM tamoxifen, and thus, this concentration was selected for further analysis. The CYP activity was induced with H_2_O_2_ (3 mM) and photosensitizer-mediated ROS produced by UV_365 nm_ (3 J/cm^2^) exposure. Controls with H_2_O_2_ and UV_365 nm_ alone or combined showed no effect on tamoxifen treated or non-treated tumor cells. First, the EPT response in the presence of tamoxifen was tested on cells treated with different nanoparticles. The untargeted P22CYP and P22CYP-PpIX showed no or little difference in the cell viability that could be attributed to the poor cellular uptake (Fig. 6a). However, targeted nanoparticles, P22CYP-PEG(EST) and P22CYP-PpIX-PEG(EST) increased the tamoxifen sensitivity by ~ twofold as depicted by the decrease in cellular viability from ~ 74 to ~ 38% (Fig. 6a). The PDT effect after UV_365 nm_ irradiation was only seen with P22CYP-PpIX-PEG(EST) and the cell suppression capacity was found similar to enzymatic prodrug treatment (~ twofold). This confirms the active targeting by estradiol derivative that resulted in the specific delivery of CYP activity and photosensitizer. The results were consistent with the cellular uptake studies. The combination of EPT and PDT using P22CYP-PpIX-PEG(EST) resulted in further decrease in viable cells to ~ 24% representing ~ threefold higher antitumor effect of tamoxifen. In addition, a double concentration of particles reduced the cell viability to ~ 16%, which is similar to the positive control (DMSO). The anti-tumor capacity of the multifunctional P22CYP-PpIXPEG(EST) showed in vitro to be highly effective for the eradication of tumor cells and an efficient therapeutic response at the lower drug dose could be expected.

The effect of PDT effect using multi-functionalized particles in the absence and presence of tamoxifen was also analyzed (Fig. 6b). In the dark environment, the different concentration of nanoparticles showed no significant difference in the cytotoxicity after 12 h of culture, portraying their intrinsic safety. Interestingly, UV_365 nm_ irradiation showed a significant difference in cytotoxicity between the tamoxifen treated and non-treated groups. The phototoxicity of P22CYP-PpIX-PEG(EST) in the absence of tamoxifen was very low (80–92% of cell viability) and concentration dependent. On the other hand, in the presence of tamoxifen, the percentage of viable cells decreased from ~ 75 to ~ 37%. This 4.5-fold enhanced PDT response suggests a role of produced ROS in the tamoxifen sensitivity. Furthermore, the extent of cytotoxicity was similar to the CYP-mediated tamoxifen transformation (EPT), as depicted by the similar anti-tumor effect (Fig. 6a). The results depict the synergy between PDT and EPT leading to enhanced cytotoxicity.

### Immunogenic response

**Fig. 7:**
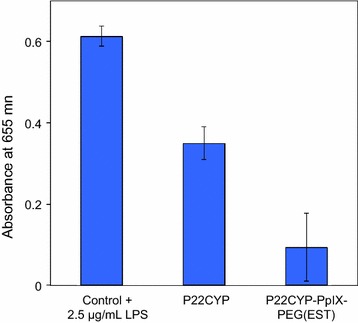
Activation of immune cells by functionalized and non functionalized VLPs was evaluated in RAW-Blue cells. RAW-Blue cells (1 × 10^5^/well) were stimulated for 12 h with medium (control), P22CYP or P22CYP-PpIXPEG(EST). In all cases, VLPs were used at a concentration of 25 μg/mL. As positive control, 21 µg protein per mL of bacterial lysate (*E. coli*) was used. Activation of the NFkB and AP-1 transcription factors was evaluated by the presence of Secreted Embryonic Alkaline Phosphatase (SEAP) by colorimetric assay ± SEM at 655 nm

The innocuousness of PEGylated (P22CYP-PpIX-PEG(EST)) and non PEGylated (P22CYP) nanoparticles was assayed on Raw-Blue cells and the amount of Secreted Embryonic Alkaline Phosphatase (SEAP) was measured. These cells express the SEAP reporter gene under the control of NF-κB and AP-1 promoters, two transcription factors that play en central role in inflammation and immunity. The non PEGylated P22CYP induced the release of SEAP in a less extent than the positive control 2.5 µg/mL of purified lipopolysaccharides from *Escherichia coli* (Fig. 7). The presence of PEG moieties on the VLP surface of P22CYP-PpIX-PEG(EST) bring down significantly the activation of NFkB and AP-1 reporter gene in macrophages. Thus the PEG cover in the multifunctional P22CYP-PpIX-PEG(EST) masks protein epitopes reducing their immunogenicity.

## Discussion

The advent of cancer nanomedicines has allowed the improvement in the narrow therapeutic window of the conventional cancer therapeutics for better survivals and enhanced treatment response. Despite the considerable technological success, there still exist major obstacles including the complex and heterogenic nature of cancers, and chemistry and manufacturing of nanomedicines for clinical translations. The often heterogenous formulations, complex surface chemistry or reproducibility limit the commercialization of nanoparticles [34, 35]. The combination nanotherapy using VLPs as nanoplatform may address the prevailing challenges. Especially, the bacteriophage capsid is a smart candidate for developing bionanomedicine due to their cost-effective production with biological uniformity [36]. Here, we introduce a multimodal platform based on P22 bacteriophage VLP integrating tumor targeting, ligand-mediated cellular internalization, delivery of CYP activity and photosensitizer payload, and the nanoscale combination of enzyme pro-drug activation therapy (EPT) and photodynamic therapy (PDT) for producing more potent, durable and highly specific anti-cancer response against ER+ breast cancer.

With a large number of surface active groups (–NH_2_, –COOH) available for multi-functionalization, VLPs enable the accommodation of a wide range of targeting moieties and photosensitizer. In the present study, PpIX was the choice of photosensitizer as it is the biological precursor of heme with the ability to be photoactivated at the Soret region (350–450 nm) or the Q-bands (500–650 nm), and sonoactivated using ultrasounds enabling deep tissue penetration [37]. The major bottlenecks for the delivery of nanotherapeutics remain the intratumoral infusion and the retention. Considering the heterogeneity of breast cancers, the better particle distribution at all the tumor sites can be assured by the systemic administration by injecting in the blood pool than the intratumoral administration. In this respect, selective targeting by enhancing the affinity of particles for tumor cells reduces the risk of toxicity to the normal cells. Therefore, an estrogen derivative, 17α-ethynylestradiol (EE2) was chosen as targeting ligand due to its high specificity towards ER+, and its synthetic accessibility. The chemical substitution at the ethynyl position of EE2 is well documented for its retained bioactivity [32, 38, 39]. Thus, chemical modification was performed at the ethynyl position of EE2 for covalent functionalization on particles surface using a heterofunctional PEG as a linker. The use of PEG also enables the control over the nonspecific cellular uptake and most importantly reducing immunogenic response of nanoparticles by providing the stealth properties [40]. The P22CYP-PpIX-PEG(EST) preparation which contains a cover of PEG molecules showed minimal activation of NFkB and AP-1 reporter gene in macrophages (Fig. 7). Several PEGylated products have been approved by the US Food and Drug Administration, European Medicines Agency, and other regulatory authorities and are clinically used with success. In addition, neither PS nor targeting ligand affected the other physical attributes of VLP after covalent conjugation. However, a relative decrease in biocatalytic activity of functionalized particles could be due to the mass transfer limitations of substrate diffusion through VLP pores due to the surface covering PEG.

P22CYP-PpIX-PEG(EST) showed active targeting and preferential uptake in ER+ cells, inducing specific delivery of CYP activity and photosensitizer payload (Fig. 3). In spite of affinity difference between the 17α-ethynylestradiol used for the nanoparticle functionalization and the 17β-estradiol, the competition experiment in the presence of free estradiol demonstrated that a ligand-receptor process governs the cell uptake (Fig. 5). The observed ligand-mediated uptake of nanoparticles suggests that the ER+ localized in plasma membrane may facilitate the selective delivery. The internalized particles showed the biocatalytic activity by actively transforming BFC to the green fluorescent HFC (Fig. 3a, c) and ability to produce ROS after UV_365 nm_ irradiation (Fig. 4). The aim of the present study is to prove the implication of VLPs for “two in one” therapeutic approach, thus, for the preliminary analysis, UV_365 nm_ (one of the high absorption wavelength of PpIX) was used as excitation source. PpIX can also be excited by high penetrating red light or ultrasound, however, the ROS production efficiency would be variable. The untargeted nanoparticles did not show efficient cellular localization or cytotoxic effects. The individual treatment of EPT or PDT in MCF-7 cells using P22CYP-PpIX-PEG(EST) enhanced the tamoxifen sensitivity up to ~ twofold (Fig. 6a). To evaluate the combinatorial treatment, the delivered enzyme and PS were activated simultaneously, which synergistically improved the tamoxifen sensitivity up to ~ threefold in vitro, leading to a strong inhibition of tumor cells.

**Fig. 8:**
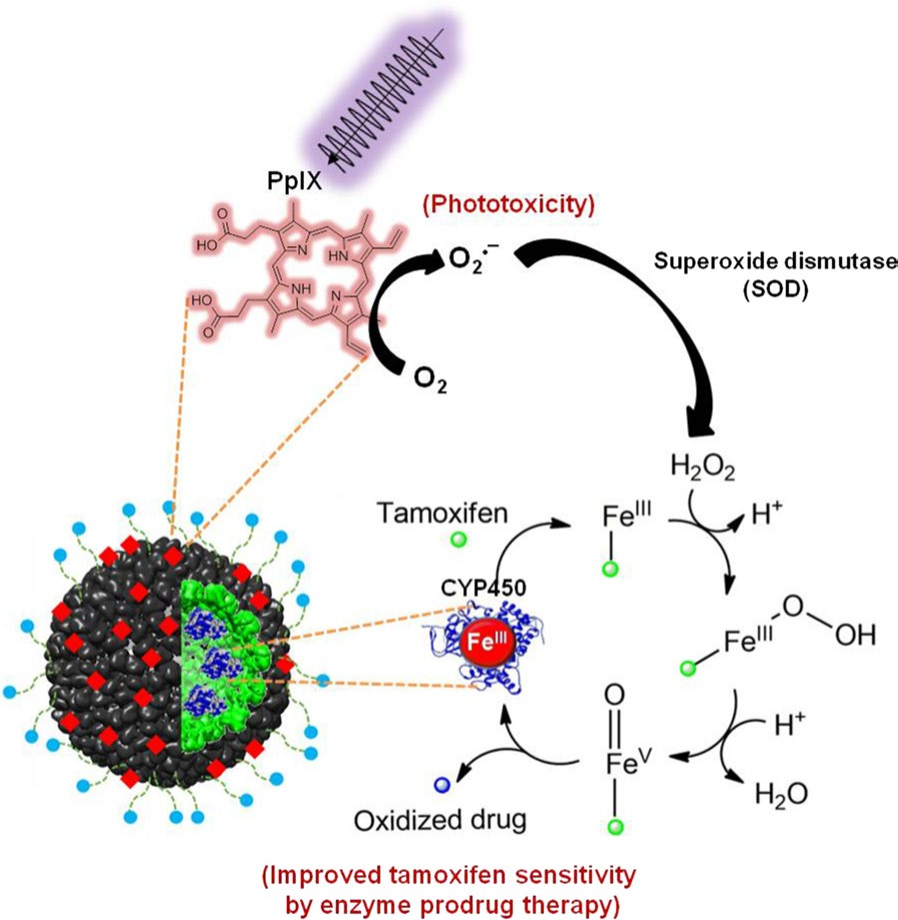
Proposed mechanism for the synergy between EPT and PDT

Without photo-simulation, P22CYP-PpIX-PEG(EST) nanoparticles were safe against MCF-7 cells tested up to 70 μg/mL. Upon activation with UV_365 nm_, the photo-toxicity of nanoparticles was very low and concentration dependent. Interestingly, this PDT response was enhanced up to ~ 4.5-fold in the presence of tamoxifen (Fig. 6b). Moreover, the extent of cytotoxicity was analogous to the CYP-mediated tamoxifen toxicity (EPT) (Fig. 6a), indicating the role of produced ROS in the tamoxifen sensitivity leading to tumor cell death. Recently, Kimakova et al. [41] stated that PDT on MCF-7 cells induces an increase of superoxide dismutase activity (SOD), which transform O_2_^·−^ radical to hydrogen peroxide causing the cells resistant to the therapy. In the developed biocatalytic VLPs, the encapsulated peroxygenases perform bioactivation of drug via H_2_O_2_-driven CYP-oxyfunctionalisation chemistry. Thus, the plausible mechanism for the synergistic effect could be the biotransformation of tamoxifen via peroxygenases driven by the production of ROS in the presence of superoxide dismutase (Fig. 8). Nevertheless, a deeper experimental analysis to elucidate this mechanism would be necessary.

## Conclusion

In conclusion, the self-assembling P22 VLP has been used to create a targeted, multivalent combinatorial nanomedicine integrating EPT and PDT for the improvement of tamoxifen treatment efficiency against ER+ breast tumors. The interior of virus capsid encapsulating CYP-activity enabled biotransformation of tamoxifen and outside multi-functionalized with PpIX and estradiol derivative enabled ROS generation upon illumination causing significant killing of ER+ cells. Thus, the use of a lower dose of the prodrug in the antitumor therapy could be expected, reducing the drastic side effects and increasing the treatment effectiveness. Interestingly, the ROS production by PS functionalized VLPs seems to be involved in the electron transfer mechanism necessary for enzymatic activation of tamoxifen and thus, making a synergistic cytotoxicity together with CYP enzymatic activity and improving the therapeutic response. PEG-coat of multifunctional VLPs renders them invisible (or stealth) for macrophages. The modular design of VLPs allows the functionalization with exchangeable PS and targeting ligands offering versatility for the other treatments requiring prodrug transformation. This innovative study shows the potential of the P22CYP-PpIX-PEG(EST) nanoreactor for further evaluation in cancer therapy.

## Methods

### Materials

The general reagents and precursors were purchased from Sigma-Aldrich (St. Louis, MO). Electrocompetent cells of *E. coli* BL21(DE3) were obtained from Lucigen (Middleton, WI). Heterofunctional polyethylene glycol with n = 4 ethylene glycol units consisting succinimidyl ester and the maleimide group (NHS-PEG_4_-Mal) was obtained from Thermo Scientific (Waltham, MA). Solvents were obtained from Sigma-Aldrich (St. Louis, MO) and used without further purification. Reactions were monitored by TLC (silica gel matrix, Sigma-Aldrich). TEM analysis was performed using copper grids (400-mesh) coated with formvar/carbon support film (TedPella, USA).

### Preparation, purification, and analysis of P22CYP VLPs

The core biocatalytic VLPs (P22CYP) were produced, purified and analyzed as previously reported [20].

### Chemical synthesis of 17-((4-aminophenyl)ethynyl)-13-methyl-7,8,9,11,12,13,14,15,16,17-decahydro-6H-cyclopenta[α]phenanthrene-3,17-diol (ESTAm)

The chemical substitution at the ethynyl position of 17α-ethynylestradiol (EE2) is well documented for its retained bioactivity [32, 38, 39]. Thus, amine functionality at the ethynyl position of EE2 was introduced by Sonogashira coupling with 4-bromoaniline to obtain ESTAm and characterized using NMR, HRMS and FTIR (Additional file 1: Figures S1–S3). Briefly, a mixture of *bis*(triphenylphosphine)palladium(II) dichloride (22 mg, 0.03 mmol) and diisopropylamine (degassed, 10 mL) were stirred for 10 min under a nitrogen atmosphere. Afterwards, copper(I)iodide (6 mg, 0.03 mmol) and 4-bromoaniline (58 mg, 0.34 mmol) were added, followed by the addition of 17α-ethynylestradiol (100 mg, 0.34 mmol). The reaction mixture was allowed to stir at 55 °C for 2 h. The brown mixture was filtered and reduced to dryness under vacuum. The crude product was dissolved in DMF (0.5 mL) and precipitated by the addition of diethylether (30 mL). The precipitate was filtered and dried to obtain desired product, ESTAm as beige color solid (93 mg, 71% yield). IR (cm^−1^, film): 3425, 2929, 2170, 1610, 1496, 1452, 1385, 1353, 1295, 1241, 1182, 1130, 1054, 1016, 917, 875, 819, 784. ^1^H NMR (400 MHz, DMSO): 8.981 (s, 1H), 7.629–7.521 (m, 4H), 7.039 (d, 1H, J = 6.8 Hz), 6.504 (dd, 1H, J = 6.8, 2.0 Hz), 6.418 (d, 1H, J = 1.6 Hz), 5.602 (s, 1H), 2.687–1.22 (18H), 0.745 (s, 3H). HR-ESI–MS: calcd for C_26_H_29_NO_2_ 387.2198, found M^+^ 387.1826, [M + H]^+^ 388.2293, [M + 2H]^+^ 389.2325.

### Surface functionalization of P22CYP with photosensitizer PpIX

The P22CYP-PpIX was synthesized by the direct conjugation of PpIX with the surface exposed amine groups of biocatalytic P22 by carbodiimide reaction [42]. The carboxylic groups of PpIX were activated to succinimidyl ester using 1-ethyl-3-(3-dimethylaminopropyl)-carbodiimide (EDC) and *N*-hydroxysuccinimide (NHS). This was subsequently reacted with P22CYP. Briefly, a solution of PpIX (6.2 μg, 11.0 nmol) in DMSO was mixed with EDC (2.4 μg, 12.77 nmol) and NHS (1.5 μg, 12.77 nmol) and kept for 30 min at room temperature (RT). It was then mixed with P22CYP (1 mg/mL) suspension in phosphate buffer (PBS) (100 mM, pH 7.4) and kept with gentle shaking for 2 h.

### Surface functionalization of P22CYP or P22CYP-PpIX with PEG-ligand moiety

For covalent conjugation of ESTAm on VLP surface, a hetero-functional polyethylene glycol (PEG) with succinimidyl ester and the maleimide group (NHS-PEG_4_-Mal) at the distal ends was used. The use of NHS-PEG_4_-Mal enabled selective conjugation of ESTAm via amide bonding giving PEG(EST), while maleimide site was free for further conjugation with nanoparticles. A solution of ESTAm (3.5 μg, 9.12 nmol) was mixed with NHS-PEG_4_-Mal (4.6 μg, 9.12 nmol) in DMSO and incubated for 1 h at RT to give PEG(EST). This was mixed with the P22CYP (1 mg/mL) and/or synthetized P22CYP-PpIX (1 mg/mL) suspension in PBS (100 mM, pH 7.4) and kept at RT with gentle shaking for 2 h. The final concentration of DMSO was less than 5% in the aqueous mixture. The particles were purified by washing with PBS via centrifugation at 8000*g* for 10 min using Amicon Ultra centrifugal filter units (Sigma-Aldrich) with 100 kDa cutoff and then resuspended in PBS (100 mM, pH 7.4).

### Characterization of nanoparticles

The morphology and size of purified nanoparticles were analyzed using transmission emission microscopy (TEM, JEOL-2010, JEOL) operated at 200 kV. The particles were negatively-stained (1% uranyl acetate) prior to TEM analysis. Dynamic light scattering (DLS) and Zeta potential measurements were performed on Zetasizer Nanoseries (Nano-ZS, Malvern Instruments). The fluorescence measurements were performed at room temperature using a Hitachi F-4700 spectrofluorometer with a 200 W Xe-lamp as an excitation source.

### DPBF assay

To detect the superoxide anion radical in the solution, we used 1,3-diphenyl-isobenzofuran (DPBF) [43]. The stock solution of DPBF (0.5 mM) in methanol was added to the samples in PBS (100 mM, pH 7.4) to obtain final concentration of 10 μM. The optical density of PpIX and P22CYP-PpIX-PEG(EST) was adjusted to 0.4 for sample preparation. The reaction mixture was irradiated with UV_365 nm_ using a UV lamp (UVL-28 EL, UVP) for different time intervals (0–30 s). The change in DPBF fluorescence was measured with excitation at 410 nm and emission at 470 nm (Additional file 1: Figure S4). DPBF itself showed a slow photodegradation in the absence of PS upon UV irradiation. By assuming the 100% conjugation of PpIX on VLP surface, we compared the results of PpIX and P22CYP-PpIX-PEG(EST) at the same overall concentration. The functionalized VLPs produced more efficient superoxide radical anion, possibly due to lack of PpIX aggregation.

### Enzymatic assay

The catalytic activity of encapsulated CYP after complete modification of particles was determined by the transformation of 2,6-dimethoxyphenol (DMP) and compared with the non-functionalized P22CYP according to our previous protocol [20, 44]. The reaction mixture (1 mL) contained DMP (500 μM) and bicatalytic VLPs (35 μg from 1 mg/mL) in PBS (100 mM, pH 7.4) and the reaction was initiated by adding H_2_O_2_ (5 mM) at RT. The extent of the reaction was monitored using an Agilent 8453 UV–Vis spectrophotometer at 468 nm (Ɛ_468_ = 14,800 M^−1^ cm^−1^).

### Cell lines and cell culture

Estrogen receptor negative (ER−) human breast adenocarcinoma MDA-MB-231 cells (HTB-26) and estrogen receptor positive (ER+) MCF-7 cells (HTB-22) were purchased from the American Type Culture Collection (ATCC). MDA-MB-231 cells were cultured in Dulbecco’s modified Eagle’s medium (DMEM) supplemented with 10% Fetal Bovine Serum (FBS, BenchMark, Gemini Bio Products), 1% penicillin streptomycin (Sigma-Aldrich), 1% l-glutamine and 1.5 g/L sodium bicarbonate. While MCF-7 cells were cultured in Eagle’s minimum essential medium (EMEM) supplemented with 10% Fetal Bovine Serum (FBS, BenchMark, Gemini Bio Products), 1% Penicillin streptomycin, 1% l-glutamine and 1.5 g/L sodium bicarbonate and 0.01 mg/mL of human recombinant insulin (Sigma-Aldrich). Cells were maintained and propagated in growth medium at 37 °C and 5% CO_2_.

### CYP enzymatic activity by confocal microscopy

The CYP activity of treated cells with targeted biocatalytic VLPs (P22CYP-PpIX-PEG(EST) and untargeted VLPs (P22CYP-PpIX) in both MCF7 and MDA-MB-231 cell lines was visualized by the transformation of 7-benzyloxy-4-trifluoromethylcoumarin (BFC) to the fluorescent product 7-hydroxy-4-(trifluoromethyl)-coumarin (HFC) according to Arora et al. [45] with some modifications. Cell culture Petri dishes coated with poly-d-lysine (MatTek P35GC1.5-10C) were used to seed 250,000 cells in DMEM media and incubated overnight at 37 °C and 5% CO_2_. The cells were incubated for 12 h at 37 °C and 5% CO_2_ with 35 μg/mL of nanoparticle preparation P22CYP-PpIX-PEG-(EST) or P22CYP-PpIX in 2 mL of cell culture media. After the incubation time, cell media was removed and the culture was rinsed with serum free DMEM-SF media. Subsequently, the BFC (15 µL, 20 mM) diluted in DMEM media (150 µL) was added to each culture plate and incubated in darkness for 10 min at RT. After this, DMEM media was added up to 1.5 mL to each plate and further incubated for 4 h at 37 °C and 5% CO_2_. Then, hydrogen peroxide (4.5 µL, 1 M) was added to each culture and incubated for 10 min at 37 °C and 5% CO_2_. Cell culture plates were rinsed three times with PBS 1×, DMEM media (2 mL) was added to each plate and incubated for 4 h at 37 °C and 5% CO_2_. BFC transformation into the fluorescent HFC product was visualized with inverted laser-scanning microscope (Olympus Fluoview, FV-100) using an argon laser for excitation at 488 nm with GFP filters for emission at 515–530 nm. P22CYP-PpIX-PEG(EST) inside the cells was visualized by the red emission of PpIX with an argon laser for excitation at 543 nm with RFP filters for emission at 655–755 nm. A plan achromatic 60 X/1.48 N.A. oil immersion objective was used. Laser intensity was kept at 20% to reduce photo bleaching. A photomultiplier module allowed the simultaneous view of fluorescence in the entire cell. Confocal images were captured using the FV-10 ASW software and were analyzed with the FV-10ASW viewer version 4.1 from Olympus. The cells were counterstained with DAPI (0.25 ng/mL) and nuclear staining was visualized with the same microscope, equipped with a LD laser for excitation at 405 nm with DAPI filters for emission at 455 nm.

### Detection of reactive oxygen species (ROS) production by confocal microscopy

The intracellular ROS generation was quantified by confocal microscopy using the DCFDA Kit from Abcam with light modifications. Cell culture Petri dishes coated with poly-d-lysine were used to seed 250,000 MCF-7 cells in DMEM media and incubated for 12 h at 37 °C in 5% CO_2_. The cultivated cells were treated with P22CYP-PpIX-PEG-(EST) or P22CYP-PpIX (35 μg/mL) and further cultured for 24 h. The cells were incubated with DCFDA (20 μM) in DMSO for 30 min at 37 °C in darkness and then exposed to UV light at 365 nm (3 J/cm^2^). The UV dose was monitored with a UV radiometer (VLX-3 W, Vilber Lourmat). The treated cells were rinsed twice with PBS (1×), fixed with 4% formaldehyde in PBS at 4 °C for 15 min. After fixation, cells were permeabilized with 0.5% Triton X-100 in PBS for 15 min at 4 °C and counterstained DAPI (0.25 ng/mL), followed by eight washes with PBS. The intracellular ROS content in MCF-7 cells treated with the particles (35 μg/mL) was analyzed on the basis of green fluorescence of DCFDA by confocal microscopy at 485 nm excitation and fluorescent emission at 535 nm.

### Competition assay

The specificity of P22CYP-PpIX-PEG(EST) to bind to MCF-7 cells was evaluated by a competition assay using two different ratios of VLP and free 17β-estradiol (0, 43 and 215 ng per μg of VLP protein) and by measuring their cell internalization by the capability to transform BFC into HFC. Briefly, MCF-7 cells (10,000 cells/well) were seeded in a 96-well plate and incubated for 24 h in cell culture media at 37 °C ad 5% CO_2_. Then, media cell culture was discarded and 5 μg of P22CYP-PpIX-PEG(EST) was added to the cells together with different amounts of free β-estradiol (0.215 and 1.075 μg). Positive control was MCF-7 cells with P22CYP-PpIX-PEG(EST) but without free 17β-estradiol and the endogenous activity of CYP was evaluated in untreated MCF-7 cells. Cells were incubated for 8 h, after which media cell culture was discarded and cells were rinsed twice. Followed by the addition of BFC (4 μL, 20 mM) diluted in DMEM media (200 μL) to each well. Cells were incubated in darkness for 1 h at 37 °C ad 5% CO_2_, after this media was discarded and cells rinsed with PBS. Then hydrogen peroxide (0.2 μL, 1 M) was added to each well and incubated for 10 min at 37 °C ad 5% CO_2_. Cells were rinsed twice and resuspended in PBS. The transformation of BFC into HFC was measured by the fluorescence intensity with a Cary Eclipse Fluorescence Spectrophotometer (Agilent) using excitation at 280 nm and excitation from 300 to 500 nm.

### Tamoxifen susceptibility assay

The increase in tamoxifen susceptibility was assayed in both cell lines as follows. A 96-well plate was used to seed 10,000 cells of MCF-7 and MDA-MB-231 cells per well and incubate them for 24 h in cell culture media at 37 °C and 5% CO_2_. Then, cell media was discarded and P22CYP-PpIX-PEG-(EST) (3.5 and 7 µg) in DMEM (100 µL) were added to each well and cells were incubated for 12 h at 37 °C and 5% CO_2_. After the incubation time, media was discarded and 50 µL of DMEM media containing H_2_O_2_ (3 mM) and tamoxifen (20 µM) was added to each well and kept for 15 min in darkness at 37 °C and 5% CO_2_. The cells were washed with DMEM media and tamoxifen (20 µM) in DMEM media (100 μL) was added to each well. Both cells were incubated for 24 h at 37 °C and 5% CO_2_. After this time, media was removed and cells were washed out with 300 µL of PBS 1× and MTT cytotoxic determination assay was achieved. Either MCF-7 or MDA-MB-231 cells without P22CYP-PpIX treatment was used as control detection for tamoxifen sensitivity. Experiments were performed in three independent replicates.

### Tamoxifen susceptibility by UV light exposure

The effect of PDT to tamoxifen treated MCF-7 cells was assessed as follows: MCF-7 (10,000/well) were seeded in a 96-well plate and incubated for 24 h in cell culture media at 37 °C and 5% CO_2_. Then, cell media was discarded, P22CYP-PpIX-PEG-(EST) (3.5 and 7 µg) in DMEM media (100 µL) were added to each well and cells were incubated for 12 h at 37 °C and 5% CO_2_. The media was discarded and DMEM media (50 µL) containing H_2_O_2_ (3 mM) and tamoxifen (20 µM) was added to each well and let it stand for 15 min at room temperature with UV exposure at 365 nm (3 J/cm^2^). After treatment, the cells were washed three times with DMEM media and tamoxifen (20 µM) in DMEM media (100 μL) was added to each well. The cells were incubated for 24 h at 37 °C and 5% CO_2_. Afterward, media was removed and cells were washed out with PBS (300 µL) and MTT cytotoxic determination assay was achieved. The cells treated with tamoxifen only were used as a control. Experiments were performed in three independent replicates.

### MTT cytotoxicity assay

After tamoxifen incubation, or UV light exposure, the viability of MCF-7 cells was tested by a colorimetric assay based on the reduction of MTT reagent (methyl-134-thiazolyl-tetrazolium) by using the TOX1 in vitro toxicology assay kit (Sigma-Aldrich). MTT reagent was added to the plate following the instructions of the manufacturer. The positive control for cell death was DMSO (dimethyl sulfoxide), which induces total cell death. Cell survival control was achieved by incubating the cells with DMEM media, simulating cell behavior under ideal conditions. Experiments were conducted independently by triplicate. Absorbance measurement of MTT reduction was achieved with a 96-well plate reader (Thermo Scientific) at 570 nm. Absorbance results from survival positive control (cell media) were used to establish 100% of cell survival, then a direct comparison of experimental groups was done and depicted as survival percentage related with tamoxifen concentration.

### Potential impact on immune response by RawBlue cells activation

The immunogenic potential of VLPs was estimated by using RAW-Blue cells (Invivogen, San Diego, CA). RAW-Blue cells are derived from murine macrophage cell line RAW 264.7. These cells express a secreted embryonic alkaline phosphatase (SEAP) reporter gene under the control of NF-κB and AP-1 promoters, two transcription factors that play en central role in inflammation and immunity. RAW-Blue cells were cultured in Dulbecco’s modified Eagle’s medium (DMEM) containing 10% fetal bovine serum (FBS) and 100 µg/mL Normocin. The cells (1 × 10^5^) were incubated with 25 μg/mL of P22CYP-PpIX-PEG(EST) or P22CYP preparations for 12 h at 37 °C and 5% CO_2_. Supernatants were collected and SEAP production was evaluated based on the activity of alkaline phosphatase (AP) after the addition of 150 µL of quantity blue and the absorbance was determined at 655 nm. As positive control, 2.5 µg of purified lipopolysaccharides from *Escherichia coli* O111:B4 (Sigma-Aldrich) was used. The experiments were carried by triplicate.

### Statistical analysis

With exception of confocal imaging acquisition, all other experiments were done in a threefold independent manner with internal triplicates. The statistical analyzes were done using GraphPad Prism v7.0 software (GraphPad Software, Inc.). The results were expressed as mean ± SEM of three independent experiments. Data were evaluated by one way ANOVA with Tukey or Dunnett’s posttest, two-way ANOVA with Tukey’s posttest and Student’s *t* test. The results were considered statistically significant when p < 0.05.

## Additional file

**Additional file 1.** Additional material on chemical analysis of synthesized estradiol derivative, ROS production kinetics and tumor cell viability vs tamoxifen concentration.

## Abbreviations

BFC: benzyloxy-4-trifluoromethylcoumarin
CYP: cytochrome P450
DCF: 2′,7′-dichloro-fluorescein
DMP: 2,6-dimethoxyphenol
DPBF: 1,3-diphenyl-isobenzofuran
EDC: 1-ethyl-3-(3-dimethylaminopropyl)-carbodiimide
EDP: enzyme prodrug therapy
ER: estrogen receptor
ESTAm: benzylamined estradiol derivative
HFC: 7-hydroxy-4-trifluoromethyl-coumarin
MTT: methyl-134-thiazolyl-tetrazolium
NHS: *N*-hydroxysuccinimide
PDT: photodynamic therapy
PEG: polyethylene glycol
P22CYP: VLPs containing cytochrome P450
P22CYP-PEG(EST): VLPs containing cytochrome P450 covered with functionalized PEG
P22CYP-PpIX-PEG(EST): VLPs containing cytochrome P450 covered with functionalized PEG and PpIX
PpIX: protoporphyrin IX
PS: photosensitizer
ROS: reactive oxygen species
SOD: superoxide dismutase
VLP: virus-like particle
