# Supplementary material for: Multifunctionalized biocatalytic P22 nanoreactor for combinatory treatment of ER+ breast cancer
Source: J Nanobiotechnology. 2018 Feb 20;16:17. doi: 10.1186/s12951-018-0345-2 (PMC5819296; doi:10.1186/s12951-018-0345-2)
Supplement: Supplementary file 1 — Additional file 1. Additional material on chemical analysis of synthesized estradiol derivative, ROS production kinetics and tumor cell viability vs tamoxifen concentration. [file 12951_2018_345_MOESM1_ESM.docx]

**Additional file 1**

**Multifunctionalized Biocatalytic P22 Nanoreactor for Combinatory Treatment of ER+ Breast Cancer**

Kanchan Chauhan, Juan M. Hernandez-Meza, Ana G. Rodríguez-Hernández, Karla Juarez-Moreno, Prakhar Sengar, and Rafael Vazquez-Duhalt^*^

Centro de Nanociencias y Nanotecnología, Universidad Nacional Autónoma de México, Ensenada, Baja California, Mexico


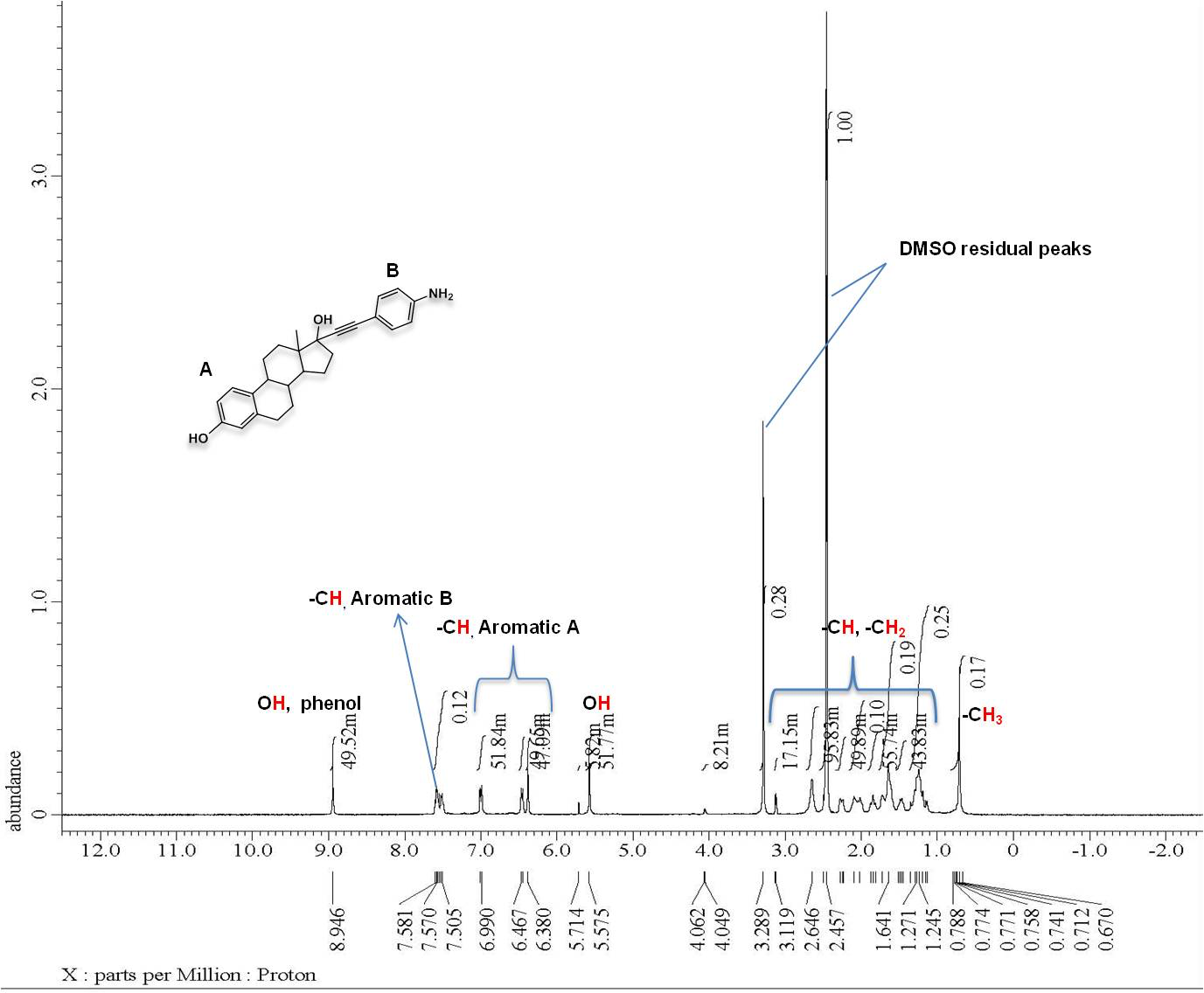


**Figure S1. ^1^H-NMR spectrum of ESTAm.** Appearance of aromatic ring B protons (δ =7.52-7.62 ppm) and disappearance of ethynyl proton (δ = 2.9 ppm) confirms the product formation.


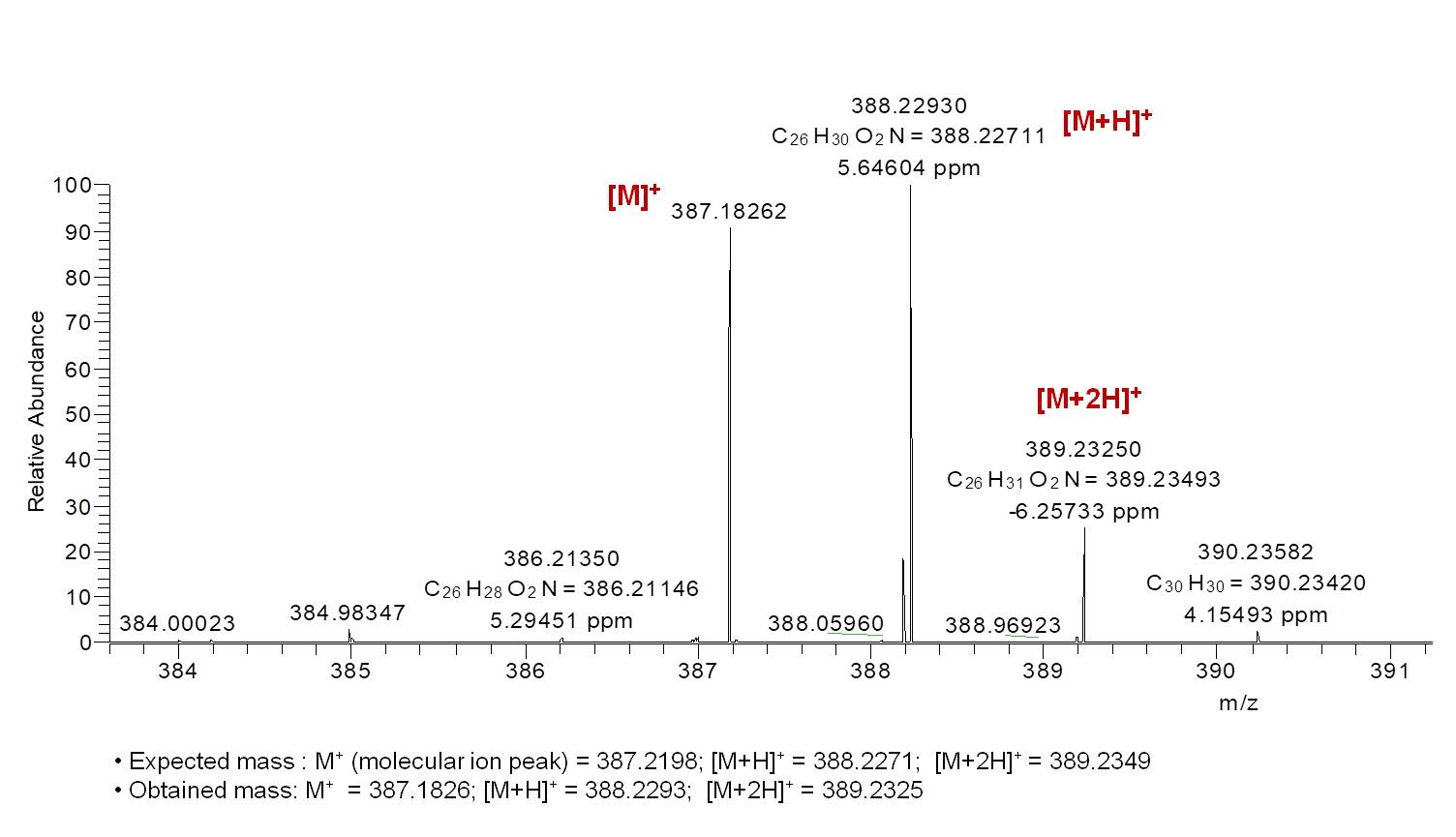


**Figure S2**. High resolution mass spectrum EstAM.


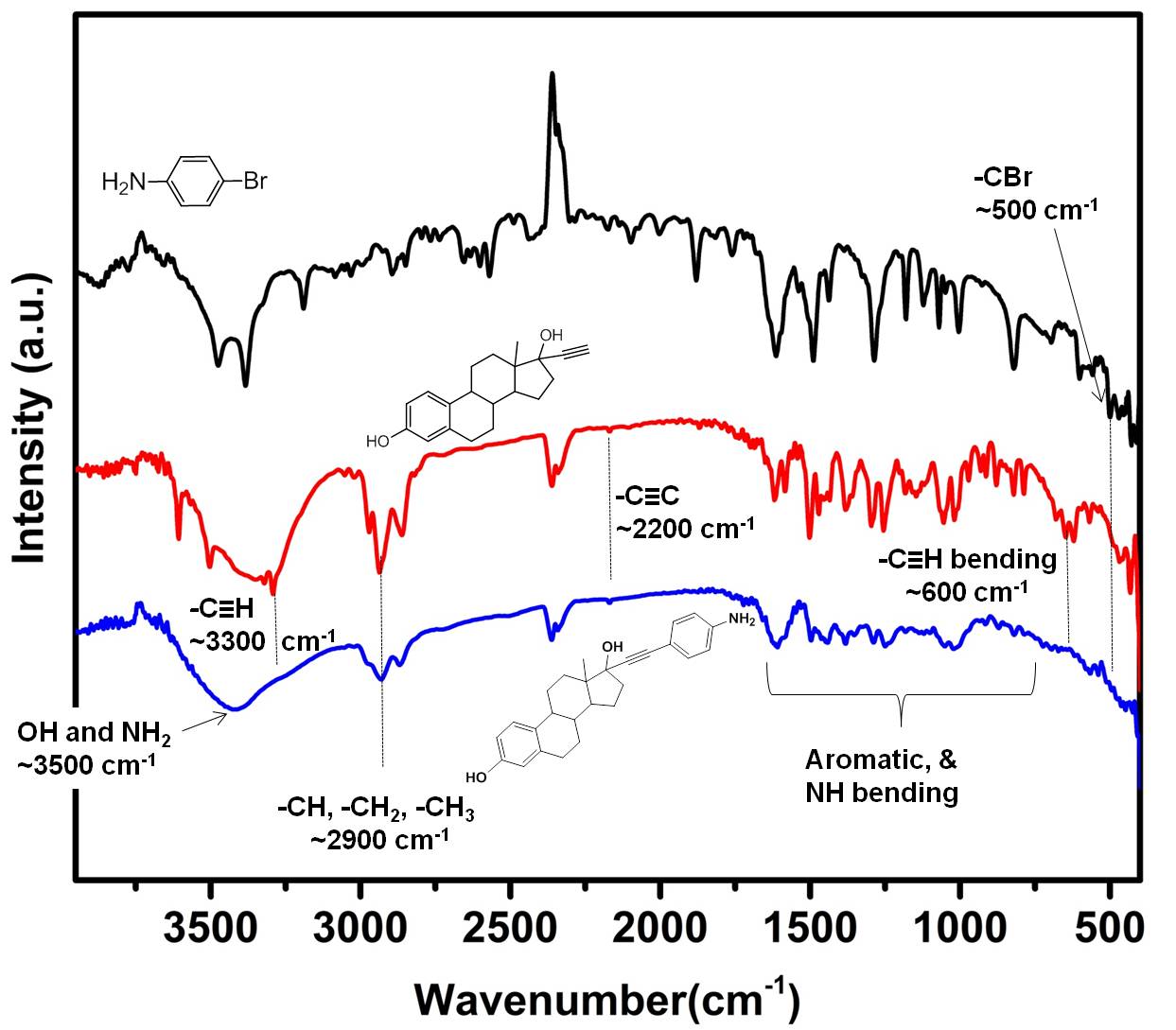


**Figure S3**. **FTIR spectrum of EstAM and the precursors.** The presence of -OH & NH_2_ stretching (~3500 cm^-1^), alkane stretching (~2900 cm^-1^), -C≡C stretching (~2200 cm^-1^), -NH bending (~1600 cm^-1^) and the absence of -C≡H stretching & bending (~3300 and 600 respectively) and –CBr stretching (~500 cm^-1^) confirms the product formation.


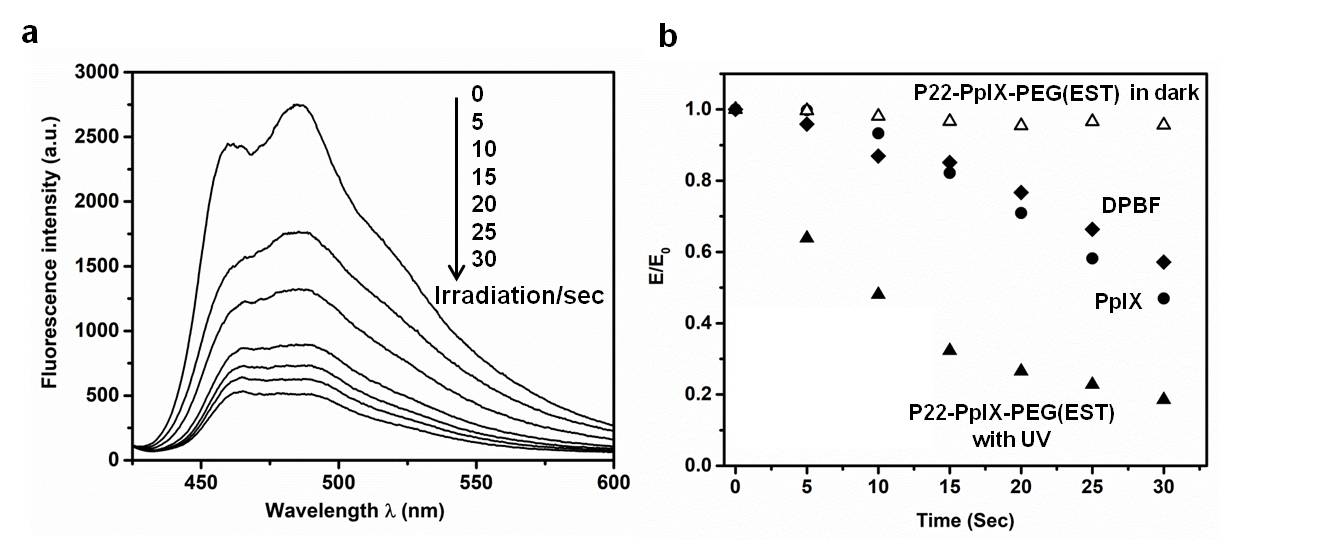


**Figure S4**. (a) Decrease in fluorescence emission of mixture of P22-PpIX-PEG(EST) and DPBF in PBS after irradiation of UV_365nm_ at different time. (b) Comparison of fluorescence intensity at λe_x_ = 410 nm of different samples mixed with DPBF: Δ P22-PpIX-PEG(EST) in dark, ▲ P22-PpIX-PEG(EST) with UV, ● PpIX with UV, ♦ PBS with UV.

The superoxide anion radical in the solution were detected using 1,3-diphenylisobenzofuran (DPBF). The stock solution of DPBF (0.5 mM) in methanol was added to the samples in PBS (100 mM, ph 7.4) to obtain final concentration of 10 μM. The optical density of PpIX and P22CYP-PpIX-PEG(EST) was adjusted to 0.4 for sample preparation. The reaction mixture was irradiated with UV_365nm_ using a UV lamp (UVL-28 EL, UVP) for different time intervals (0-30 sec). The change in DPBF fluorescence was measured with excitation at 410 nm and emission at 470 nm. DPBF itself showed a slow photodegradation in the absence of PS upon UV_365nm_ irradiation. The mixture of DPBF and P22CYP-PpIX-PEG(EST) reached the saturation after only 30 s irradiation time. The direct comparison of PpIX and the functionalized VLPs at the same overall PpIX concentartion shows the VLPs to be more efficient in the production of superoxide radical anions, possibly due to lack of PpIX aggregation.

**Figure S5**. Cellular viability of MCF-7 cells after treatment with different concentrations of tamoxifen. Results are expressed as the mean ± SEM (n=3).
